# Artificial intelligence-based computational framework for drug-target prioritization and inference of novel repositionable drugs for Alzheimer’s disease

**DOI:** 10.1186/s13195-021-00826-3

**Published:** 2021-05-03

**Authors:** Shingo Tsuji, Takeshi Hase, Ayako Yachie-Kinoshita, Taiko Nishino, Samik Ghosh, Masataka Kikuchi, Kazuro Shimokawa, Hiroyuki Aburatani, Hiroaki Kitano, Hiroshi Tanaka

**Affiliations:** 1 Research Center for Advanced Science and Technology, The University of Tokyo, 4-6-1 Komaba, Meguro-ku, Tokyo, 153-8904 Japan; 2 The Systems Biology Institute, Saisei Ikedayama Bldg. 5-10-25 Higashi Gotanda Shinagawa, Tokyo, 141-0022 Japan; 3 Institute of Education, Tokyo Medical and Dental University, 20F, M&D Tower, 1-5-45 Yushima, Bunkyo-ku, Tokyo, 113-8510 Japan; 4 SBX BioSciences, Inc, 1600 - 925 West Georgia Street, Vancouver, BC V6C 3L2 Canada; 5 Faculty of Pharmacy, Keio University, 1-5-30 Shibakoen, Minato-ku, Tokyo, 105-8512 Japan; 6 Department of Genome Informatics, Graduate School of Medicine, Osaka University, 2-2 Yamadaoka, Suita, Osaka, 565-0871 Japan; 7 Center for Mathematical Modeling and Data Science, Osaka University, 1-3 Machikaneyama-cho, Toyonaka City, Osaka, 560-8531 Japan

**Keywords:** Network embedding, Deep learning, Machine learning, Systems biology, Drug discovery, Protein interaction network

## Abstract

**Background:**

Identifying novel therapeutic targets is crucial for the successful development of drugs. However, the cost to experimentally identify therapeutic targets is huge and only approximately 400 genes are targets for FDA-approved drugs. As a result, it is inevitable to develop powerful computational tools that can identify potential novel therapeutic targets. Fortunately, the human protein-protein interaction network (PIN) could be a useful resource to achieve this objective.

**Methods:**

In this study, we developed a deep learning-based computational framework that extracts low-dimensional representations of high-dimensional PIN data. Our computational framework uses latent features and state-of-the-art machine learning techniques to infer potential drug target genes.

**Results:**

We applied our computational framework to prioritize novel putative target genes for Alzheimer’s disease and successfully identified key genes that may serve as novel therapeutic targets (e.g., DLG4, EGFR, RAC1, SYK, PTK2B, SOCS1). Furthermore, based on these putative targets, we could infer repositionable candidate-compounds for the disease (e.g., tamoxifen, bosutinib, and dasatinib).

**Conclusions:**

Our deep learning-based computational framework could be a powerful tool to efficiently prioritize new therapeutic targets and enhance the drug repositioning strategy.

**Supplementary Information:**

The online version contains supplementary material available at (10.1186/s13195-021-00826-3).

## Background

Biomedical research, especially for the field of drug discovery, is currently experiencing a global paradigm shift with artificial intelligence (AI) technologies and their application to “Big Data” in the biomedical domain [1–3]. The complex, non-linear, multi-dimensional nature of big data is accompanied by unique challenges and opportunities when employed for processing and analysis to derive actionable insights. In particular, existing statistical techniques, such as principle components analysis (PCA), are insufficient for capturing the complex interaction patterns that are hidden in multiple dimensions across the data spectrum [4]. Thus, a key challenge for future drug discovery research is the development of powerful AI-based computational tools that can capture multiple dimension of biomedical insights and obtain “value” in the form of actionable insights (e.g., insights toward to select and prioritize candidate targets and repositionable drugs for candidate targets) from big data volumes.

“Big Data” in the biomedical domain are generally associated with high dimensionality. Their dimensionality should be reduced to avoid undesired properties of high-dimensional space, such as the curse of dimensionality [5]. Dimensionality reduction techniques facilitate classification, data visualization, and high-dimensional data compression [6]. However, classical dimensional reduction techniques (e.g., PCA) are generally linear techniques and thus insufficient to handle non-linear data [4, 6].

With the recent advancement in AI technologies, several dimensionality reduction techniques have become available for non-linear complex data [4, 6, 7]. Among the dimensionality reduction techniques, the multi-layer neural network-based technique, “deep autoencoder,” could serve as the most powerful technique for reducing the dimensionality of non-linear data [4, 6]. Deep autoencoders are composed of multilayer “encoder” and “decoder” networks. The multilayer “encoder” component transforms high-dimensionality data to a low-dimensional representation while multilayer “decoder” component recovers original high-dimensional data from the low-dimensional representation. Weights associated with the links that connect the layers are optimized by minimizing the discrepancy between the input and output of the network (i.e., in an ideal condition, the values for the nodes in the input layer is the same as those in the output layer). After the optimization steps, the middle-hidden encoder layer yields a low dimensional representation that preserves information that is considered original data as much as possible [6]. The values of nodes in the middle-hidden encoder layer would be useful features for classification, regression, and data visualization of high-dimensional data.

In drug discovery research, identifying novel drug-targets is critical for the successful development of a therapeutic drug [8–10]. However, the cost to experimentally predict drug targets is huge and only approximately 400 genes are used as targets of FDA-approved drugs [11]. Thus, it is inevitable to develop a powerful computational framework that can identify potentially novel drug-targets.

Drug repositioning is another promising approach for boosting new drug development. The advantage of drug repositioning is its established safety (i.e., toxicology studies have already been carried out with a target drug). Therefore, the development of computational methods to predict repositionable candidates could be a promising strategy to reduce the cost and time for drug development.

Different drug repositioning methods have been proposed in prior studies. Further, these methods can be classified into two different major categories: activity-based drug repositioning and in silico drug repositioning. Several drugs for non-cancerous diseases have been discovered for cancer therapeutics using the former approach [12], and in recent years, the latter approach has become successful because of advancements of the protein-protein interaction database, protein structural database, and in-silico network analysis technology. Such types of applications for drug repositioning via the network theory have also been discussed. By verifying the similarity between CDK2 inhibitors and topoisomerase inhibitors, Iorioet et al. [13] reported that Fasudil (a Rho-kinase inhibitor) might be applicable to several neurodegenerative disorders. Further, Cheng et al. [14] applied the inference method based on three similarities (drug-based, target-based, and network-based similarities) to predict the interactions between drugs and targets and finally confirmed that five old drugs could be repositioned.

PIN data could be a useful resource for computational investigations of potential novel drug-targets; that is because proteins derive their functions together with their interacting partners and a network of protein interaction captures downstream relationships between targets and proteins [8–10, 15]. With the recent advancement in network science, various network metrics are presently available and have been used to investigate the structure of molecular interaction networks and their relationship with drug-target genes [8–10, 15, 16]. For example, “degree,” which is the number of links to a protein, is a representative network metric for investigating the molecular interaction networks (i.e., almost all FDA-approved drug-targets are middle- or low-degree proteins; however, almost no therapeutic targets exist among high-degree proteins [10]). Such finding indicates that the key features for identifying potential drug target genes could be embedded in the complex architectures of the PIN [10].

Genome-wide PIN data are typical non-linear high-dimensional big-data in the biomedical domain that are composed of thousands of proteins as well as more than ten-thousand interactions among them [8, 9]. Mathematically, a PIN is represented as an adjacency matrix [17]. The adjacency matrices for PINs within rows and columns labeled by proteins and elements in the matrices are presented as a binary value (i.e., 1 or 0 in position (*i,j*) if protein *i* interacts with protein *j* or not). In the adjacency matrix, each row represents the interacting pattern for each protein and may be a useful feature for predicting potential drug target proteins.

Recently, researchers have developed “network embedding” methods that apply dimensional reduction techniques to extract low-dimensional representations of a large network from the high-dimensional adjacency matrix of the network [17, 18]. For example, several researchers have used singular value decomposition and non-negative matrix factorization methods to map high-dimensional adjacency matrices of large-scale networks onto low-dimensional representations [19, 20]. However, the feature vector for a protein is high dimensional (e.g., several thousand dimensions) and sparse; this is because protein interaction network composed of thousands of proteins and the vast majority of proteins in the PIN have few interactions [17].

To address this issue, several researchers have employed network embedding methods based on deep learning techniques [21, 22]. Deep autoencoder-based network embedding methods would be especially useful for transforming non-linear large-scale networks into low-dimensional representations. Wang et al. applied a deep autoencoder-based network embedding method to large-scale social networks (e.g., arxiv-GrQc, blogcatalog, Flicker, and Youtube) and successfully mapped these networks onto low-dimensional representations [21].

Herein, to infer potentially novel target genes, we proposed a computational framework based on a representative network embedding method that employs a deep autoencoder to map a genome-wide protein interaction network onto low-dimensional representations. The framework builds a classifier based on state-of-the-art machine learning techniques to predict potentially novel drug-targets using the resultant low-dimensional representations. We applied the framework to predict potentially novel drug targets for Alzheimer’s disease. Based on the list of predicted candidate novel drug targets, we further inferred potential repositionable drug candidates for Alzheimer’s disease.

## Methods

### Overview

The first part of the method was preparing the PIN data and calculating the 100 dimension vector representation for each gene by using a deep autoencoder. To examine the performance of the deep autoencoder, we compared the 100 features with nine known network metrics. The second part was building a machine learning model which can predict whether a gene is a putative target of Alzheimer’s therapeutic drug or not. In this step, we used Xgboost to build the model and SMOTE to mitigate the sample imbalance (i.e. only a few genes were known therapeutic targets).

### PIN data and drug-target information

The PIN data was obtained elsewhere [23]. This network is composed of 6,338 genes and 34,814 non-redundant interactions among the genes.

We obtained information for drugs and their target genes from the DrugBank database [24, 25]. Thereafter, we investigated the “description” field for all the drugs in the DrugBank database and identified 61 therapeutic drugs for Alzheimer’s disease. The 61 targets for these drugs were regarded as the established drug targets for Alzheimer’s disease. Among the 61 targets, 31 were mapped onto the PIN.

### Feature extraction from PIN using a deep autoencoder

We build a deep autoencoder with a symmetric layer structure composed of 7 encoder layers and 7 decoder layers (e.g., 7 encoder layers (6338-3000-1500-500-250-150-100) and symmetric decoder layers (100-150-250-500-1500-3000-6338)). Layers are fully connected. In addition, layers, except output layer, use rectified linear unit (ReLU) [26] as an activation function while output layer uses sigmoid function to generate binary outputs. We optimized the deep autoencoder network by using “adam” [27] optimizer with a learning rate =1.0×10^−6^, number of epochs = 10,000, batch size = 10, and default values for the other parameters. In the optimization step, we minimized the binary cross-entropy loss between the values of nodes in the input layer and those in the output layer. We used a representative deep learning platform, “Keras” [28], with Tensorflow [29] backend to implement the deep autoencoder. To perform the deep autoencoder-based dimensionality reduction analysis of PIN, we used Tesla K80 GPU on the shirokane 5 super computer system [30].

### Statistical and topological analysis of the PIN

To determine the statistical topological features in the PIN for each gene, we calculated the following representative network metrics: indegree, outdegree, betweenness, closeness, PageRank [31], cluster coefficient [32], nearest neighbor degree (NND) [33], bow-tie structures [34], and indispensable nodes [35, 36].

Indegree: Indegree for a given node represents the number of nodes connected to the node (i.e., upstream neighbors of the node).

Outderee: Outdegree represents the number of links from the given node to other nodes (i.e., downstream neighbors of the nodes).

Betweenness: Betweenness for a given node *i* is the number of shortest paths between two nodes that pass through node *i*.

Closeness: The value of closeness for a given node *i* is the mean length of the shortest paths between node *i* and all other nodes in the network.

PageRank [31]: PageRank for a given node is a metric used to roughly estimate the importance of the node in the network. The PageRank score is calculated using the algorithm proposed by Google [37]. A given node has a higher PageRank if the nodes with a higher rank have links to the node.

Cluster coefficient [32]: Cluster coefficient of a node *i* (*C*_*i*_) is calculated by using the following equation: $C_{i} = \frac {2e_{i}}{k_{i}(k_{i}-1)}$, where *k*_*i*_ is the degree of node *i* and *e*_*i*_ is the number of links connecting the neighborhood of node *i* to one another.

Nearest neighbor degree (NND) [33]: The value of NND for a given node *i* is the average degree among nearest neighbor nodes of node *i*.

Bow-tie structure [34]: Biological networks often possess bow-tie structures that are composed of three components (i.e., input, core, and output layers). Yang et al. proposed a bow-tie decomposition method to classify nodes into three classes: the input layer, the core layer, and the output layer [34]. In the decomposition analysis, a strongly connected component composed of the largest number of nodes is defined as the nodes in the core layer. Nodes in the input layers can reach the core layer; however, those in the core layer cannot reach the input layer. Further, the nodes in the core layer can reach the nodes in the output layers but those in the output layer cannot reach the core layer. Herein, one-hot vector encoding was employed to represent the analysis results from bow-tie decomposition. For example, for a node assigned to the core layer, the value of the “core layer” of the node is equal to 1 while the value of the “input layer” and the “output layer” is equal to 0.

Indispensable nodes [35, 36]: Liu et al. developed a controllability analysis method to identify the minimum number of driver nodes (ND) that must be controlled to modulate the dynamics of the entire network [36] (i.e., they used the Hopcroft–Karp “maximum matching” algorithm [38] to identify the minimum set of driver nodes [36]). Indispensable nodes that are potential key player nodes and are sensitive to structural changes in a network are obtained from controllability analysis (i.e., removal of an indispensable node increases the ND in the network [35]). Vinayagam et al. reported that indispensable proteins in the human PIN tend to be targets of mutations associated with human diseases and human viruses [35]. One-hot vector encoding was also used to represent the analysis results of indispensable nodes. For example, for an indispensable node, the value of the binary variable of the node is equal to 1 while that for a non-indispensable node is equal to 0.

For network analysis, we employed the igraph R package [39].

### Oversampling by the SMOTE algorithm

In order to prepare a class-balanced dataset for building binary classifier, we used a state-of-the-art sampling method, SMORT [40], to generate this class-balanced dataset to construct a binary classifier for drug target prediction. The SMOTE algorithm synthetically creates more cases in the minority class. Thus, the algorithm selects k nearest neighbours of a case in the minority class and randomly selects a point along the line that connects them. The selected point is used as an additional case in the minority class. We used the Python module, imbalance-learn[41], to perform oversampling based on the SMOTE algorithm. In addition, we used *k*=2 to carry out SMOTE-based oversampling.

### Binary classifier model based on Xgboost

To build a binary classifier for drug target prediction, we used Xgboost, which is the most efficient implementation of the gradient tree boosting algorithms [42]. The algorithm generates a large number of weak learners and builds a strong learner that exists as an ensemble of the weak learners. In the boosting step, the algorithm continues to update the weak learners by correcting the errors made by previous learners. Thereafter, the algorithm aggregates the predictions from the weak learners to make the final prediction by minimizing the loss with a gradient descent algorithm.

To build the Xgboost algorithm-based binary classifiers, we used the XGBClassifier and scikit-learn [43] python modules. The XGBClassifier has several parameters. Briefly, we employed the following values for each parameter (please see manual for XGBClassifier module [44] for details): learning_rate = (0.01, 0.1,0.5), max_depth = (1, 2, 3, 5, 10), n_estimators = (100), gamma = (0, 0.3), boostor = (“gblinear”), objective = (“binary:logistic”), reg_lambda = (0, 0.1, 1.0), and reg_alpha = (0, 0.1,1). For the other parameters, we used a default value. To evaluate the binary classifier models and optimize the parameters of the models, we performed 5-fold cross validation.

### Pathway enrichment analysis

To identify the pathways that are significantly associated with the putative targets inferred by our computational framework, we used WebGestalt web tool [45]. WebGestalt uses over-representation analysis (ORA) to statistically evaluate overlaps between the gene set of interest and a pathway [46]. In the analysis, the number of overlapped genes between the gene set of interest and a pathway is first counted. Thereafter, a hyper-geometric test is used to determine whether the pathway is over- or under-represented in the gene set of interest (for each pathway, the *p* value and FDR are calculated based on the overlap). Based on the ORA, we examined the pathways in Reactome, KEGG, and GO biological processes. The pathways with an *FDR*<0.05 were regarded as significant pathways associated with the gene set of interest.

## Results

### Network embedding: deep autoencoder-based dimensional reduction of PIN

We obtained the directed human PIN from [23]; this PIN is composed of 6338 genes and 34,814 interactions (see the “Methods” section for details). Thereafter we generated an adjacency matrix for the human PIN. Elements in the matrix are represented as a binary value (i.e., 1 or 0 in position (*i,j*) denotes whether or not protein *j* is a downstream interacting partner of protein *i*). The resultant matrix is composed of 6,338 rows and 6,338 columns. Each row in the matrix presents the interacting pattern for each gene and used as features of the gene. Because there are 6,338 genes in the PIN, the features for each gene are of 6,338 dimensions (i.e., a gene is characterized by 6338 dimensional features based on the PIN data).

**Fig. 1:**
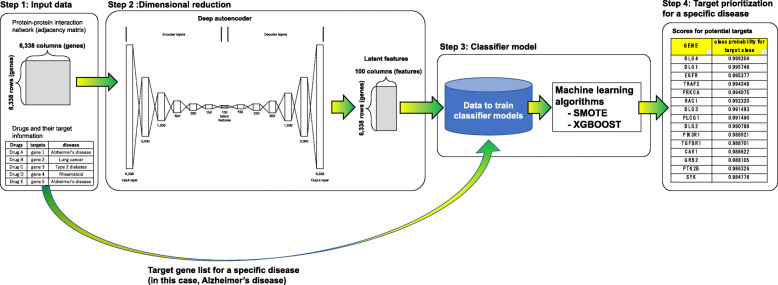
Computational analysis pipeline for drug target prioritization. (Step 1) Our computational framework employed genome-wide PINs and information of drug targets obtained from public domain databases. (Step 2) The framework is based on a deep autoencoder to extract low-dimensional latent features from high-dimensional PIN. (Step 3) By using features from step 2 and a target gene list for a specific disease, we generated 100 datasets to train the 100 classifier models. By using the 100 datasets and the state-of-the-art machine learning techniques (SMOTE and Xgboost), we build 100 classifier models to infer potential drug targets. (Step 4) We applied the classifier models to all unknown drug-target genes in the PIN to prioritize potential drug target genes

As shown in Fig. 1, to map the high dimensionality of the features (6338 dimensions) for each gene onto low dimensional features, we built and used a deep autoencoder. The deep autoencoder is composed of 7 encoder layers (6338-3000-1500-500-250-150-100) and symmetric decoder layers (100-150-250-500-1500-3000-6338) (see Fig. 1). In the deep autoencoder, layers are fully connected and weights of links connecting layers are optimized by minimizing binary cross-entropy loss between values of nodes in the input layer and those in the output layer (for details, see the “Methods” section). Following optimization, for each gene, we used the optimized deep autoencoder to map the high dimensionality of the original features (6,338 dimensional features) into low dimensionality (100 dimensional features) through the middle layer (layer with 100 nodes) in the network. Accordingly the resultant features for each gene are of 100-dimensional features.

**Fig. 2:**
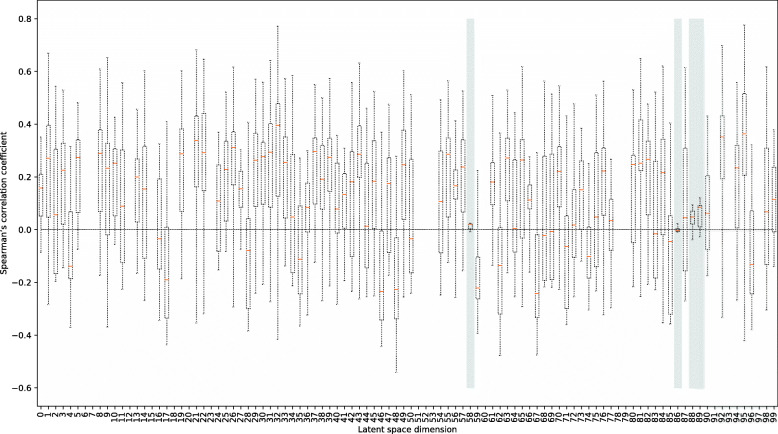
Relationship between features in low-dimensional latent space by deep autoencoder and representative network metrics in the PIN. The *X*-axis is the latent space dimension and the *Y*-axis is Spearman’s correlation coefficient between a given low-dimensional feature and a given network metric (see Supplementary Figure 1 for the original data). The gray background dimensions (58, 86, 88, and 89) indicate almost no correlation to the representative network metrics. Several dimensions without the box (e.g., dimension 6 and 7) are n.a. because the encoded numerical values for all genes are zero

The low-dimensional latent space contains enough information to represent original high-dimensional human PIN. However, it is still unclear whether the low-dimensional features in the latent scape can explain the topological and statistical properties obtained from the representative network metrics. To examine this issue, we calculated nine representative network metrics for each gene in the PIN (e.g., indegree, outdegree, betweenness, closeness, PageRank, cluster coefficient, nearest neighbor degree (NND), bow-tie structure, and node dispensability, see the “Methods” section for details) and compared the metrics to the 100-dimensional features for the gene from the network embedding analysis (see Fig. 2 and the original data for Supplementary Figure 1). As shown in the figure, among the 100-dimensional feature, most of the features were correlated with the representative network metrics. Interestingly, several features (e.g., dimensions 58, 86, 88, and 89) did not correlate with the nine representative network-metrics (shown in gray background). Such findings indicate that the low-dimensional features from the network embedding analysis can capture not only the topological and statistical properties of network metrics but also information that cannot be obtained from analysis using representative network metrics.

### Machine learning-based drug target prediction using the extracted feature from PIN

In this study, we treated the issue of drug-target prediction as a binary classification model. To construct a binary classifier for drug-target prediction, we generated a training dataset using the low-dimensional features extracted from PIN and public domain drug-target information. From the public domain drug-target database, we obtained known drug-target genes for Alzheimer’s disease. Among the known targets, we could map 31 onto PIN. These 31 genes were further regarded as positive cases and the negative cases were selected from the remaining 6,307 genes. We randomly selected 500 negative cases (genes) from the 6307 genes 100 times to build 100 datasets composed of 500 negative and 31 positive cases (genes). In the 100 datasets, each gene had 100 dimensional features that were obtained from deep autoencoder. Further, we employed the 100 datasets to build 100 binary classifier models to predict novel candidate targets for Alzheimer’s disease.

The 100 datasets are class-imbalanced (e.g., 31 positive and 500 negative cases, respectively). Furthermore, classification using class-imbalanced data is biased toward the majority class. In the datasets, the number of “positive” cases was very small (i.e., only 31 positive cases were found in the datasets). These problems can be mitigated by using over-samplings that are often used to produce class-balanced training datasets from class-imbalance data. To generate class-balanced training datasets for binary classifiers, we used a state-of-the-art sampling method, SMOTE (Synthetic Minority Oversampling TEchnique) [40] that synthetically creates new cases in the minority class (in this study, “positive” case) (see the “Methods” section in details).

By using the class-balanced training datasets from SMOTE, we trained binary classifiers for drug target prediction. The binary classifier models are based on the Xgboost algorithm which is the most efficient implementation of the gradient boosting algorithm [42]. The trained binary classifier models calculate two class probabilities for each gene based on 100 dimensional features (e.g., probability of “positive” and that of “negative”). Accordingly, a gene with a higher class probability of “positive” is more likely to be a member of the “positive” class.

To optimize the binary classifiers based on Xgboost for drug target prediction, we performed a grid search with 5-fold cross validations. Notably to avoid data leakage, we conducted data splits for cross validations before SMOTE-based over-sampling to generate class balancing training datasets. To evaluate the predictive performance of each parameter combination, we calculated area under the receiver operator characteristic curve (AUC ROC). The mean value of AUC ROC for the 100 binary classifiers with the optimal parameters was 0.661. Such result indicates that the 100 binary classifiers tend to assign a high class probability of “positive” for known drug-target genes of Alzheimer’s disease. Therefore, unknown drug-target genes with a high probability of “positive” could serve as novel drug-targets for Alzheimer’s disease.

Further, to infer the putative therapeutic targets for Alzheimer’s disease, we used the mean value of the class probability of “positive” from the 100 binary classifier to prioritize the 6,307 genes (see Table 1 and Supplementary Table 1 for details); i.e., the unknown targets with a higher mean value of “positive” for the class probability (e.g., DLG4 in Table 1 and Supplementary Table 1) are more likely potential novel drug targets. A total of 187 unknown drug-target genes had a mean value greater than 0.75 for a class probability of “positive” (see Supplementary Table 1). These 187 genes were thus regarded as putative novel target genes for Alzheimer’s disease.

**Table 1:** Top 20 genes with the highest mean probability value for the “positive (drug target)” class

**Gene**	**Mean probability**
DLG4	0.99859
PLCG1	0.99775
EGFR	0.99758
SYK	0.99752
PTK2B	0.99617
RAC1	0.99585
CAV1	0.99579
DLG1	0.99512
PIK3R1	0.99500
PRKCA	0.99292
KIT	0.99224
JAK1	0.99154
PTPN6	0.98968
CRKL	0.98918
SHC1	0.98840
NCK1	0.98760
ZAP70	0.98750
PTPN11	0.98630
DLG3	0.98551
PTK2	0.98537
DLG2	0.98471
IL2RB	0.98328
JAK2	0.98299
GRB2	0.98278

### Pathway enrichment analysis of putative target genes

**Fig. 3:**
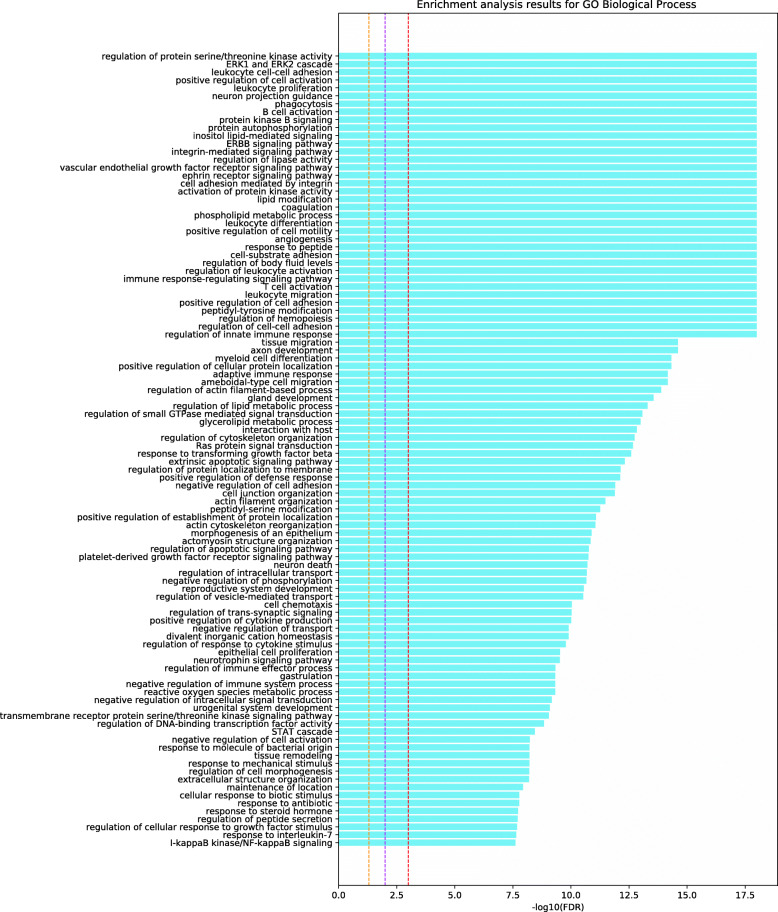
Pathway enrichment analysis using GO biological database for the 187 putative targets from our computational pipeline for Alzheimer’s disease. The names of the pathways are shown on the vertical axis, and the bars on the horizontal axis represent the − log10(*p* value) of the corresponding pathway. Dashed lines in orange, magenta, and red indicate *p* value <0.05, 0.01, and 0.001, respectively

**Fig. 4:**
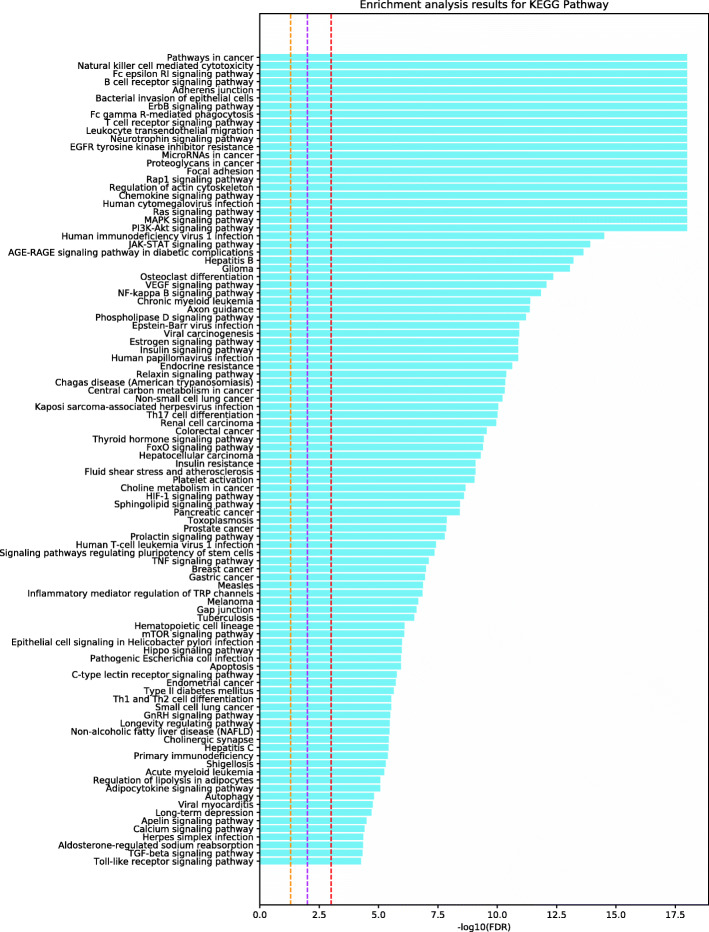
Pathway enrichment analysis using the KEGG database for 187 putative targets. The legend for this figure is the same as that for Fig. 3

**Fig. 5:**
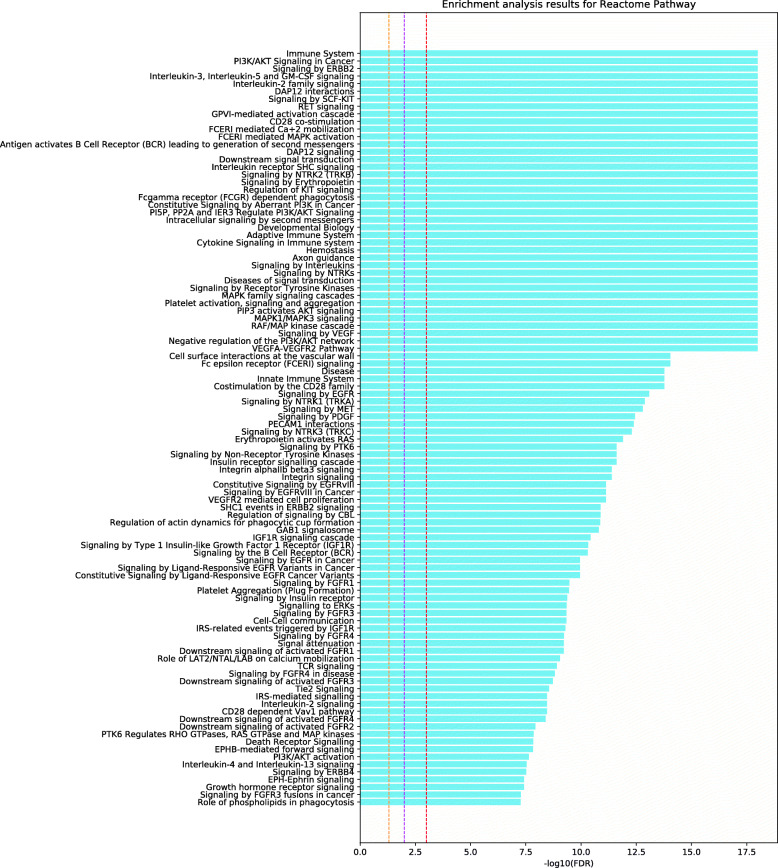
Pathway enrichment analysis using the Reactome pathway for 187 putative targets. The legend for this figure is the same as that for Fig. 3

To deduce the potential target pathways for Alzheimer’s disease, we determined the significant pathways that are associated with the 187 putative targets inferred using our computational framework (see Figs. 3, 4, and 5). The 187 putative targets were significantly associated with the pathways that control Alzheimer’s disease mechanisms (e.g., cytokine-related signaling pathways and EGF receptor signaling pathway), especially those associated with inflammatory mechanisms and the immune system. The innate immune system is a key component of Alzheimer’s disease pathology [47]. In fact, continuous amyloid- *β* formation and deposition chronically activate the immune system, causing disruption of the microglial clearance systems [47]. Accordingly, the progression of Alzheimer’s disease could be suppressed by modulating these pathways, especially the immune system and inflammation-related pathways, by targeting these putative target genes.

### Inference of repositionable drug candidates

**Fig. 6:**
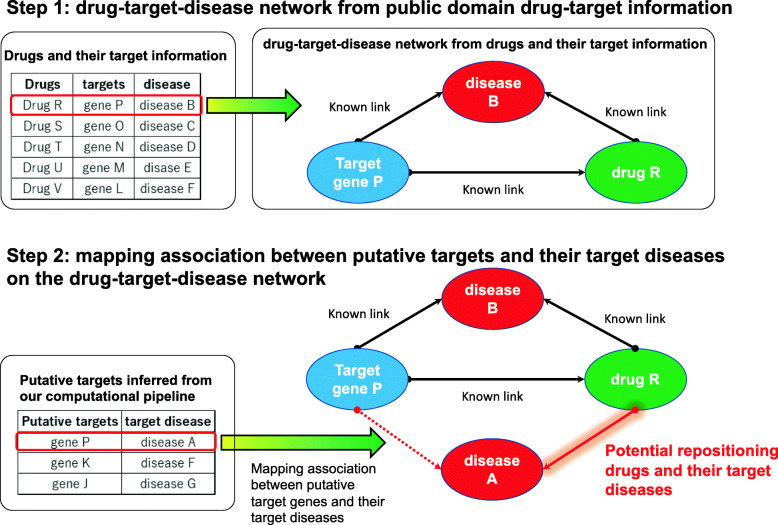
A method to infer potential repositionable drugs based on the putative targets derived from our computational pipeline. Step 1: We obtained the drug-target-disease network from the DrugBank database. Step 2: We mapped the associations between the putative target genes and their target diseases to infer the potential repositionable drugs for a given disease

Networks connecting drugs, targets, and diseases could serve as useful resources for investigating novel indications for FDA-approved drugs, i.e., if target gene P is a putative target for disease A and is a known target gene of drug R for disease B, disease A may be a novel target disease for drug R (see Fig. 6). Thus, to infer the putative repositionable drugs and their potential target disease, we further examined the list of 187 predicted putative target genes (genes with a class probability of target class >0.75 in Supplementary Table 1) from our computational framework and drug-target information across different diseases. If at least one target of an known drug is included among the 187 putative targets, the drug was regarded as a potential repositionable drug. As shown in Supplementary Table 2, we inferred 244 candidate repositionable drugs for Alzheimer’s disease. For each candidate repositionable drug, we calculated the number of overlapping genes between the known targets of the drug and the 187 putative targets. Thereafter, we ranked the candidate repositionable drugs based on the number of overlapped genes. Among the predicted repositionable drug candidate, the top ranked candidates may be effective for the target disease. Table 2 lists the 20 highest ranked candidate compounds.

**Table 2:** Top 20 candidate repositioning drugs for Alzheimer’s disease

DRUG	Overlaps between known targets and predicted targets	# of overlaps
Regorafenib	RET; FLT1; KDR; KIT; PDGFRA; PDGFRB; FGFR1; TEK; NTRK1; EPHA2; ABL1	11
Tamoxifen	ESR1; ESR2; PRKCA; PRKCB; PRKCD; PRKCE; PRKCG; PRKCQ; PRKCZ; ESRRG	10
Ponatinib	ABL1; KIT; RET; TEK; FGFR1; LCK; SRC; LYN; KDR; PDGFRA	10
Dasatinib	ABL1; SRC; FYN; LCK; KIT; PDGFRB; EPHA2; BTK; FGR; LYN	10
Imatinib	PDGFRB; ABL1; KIT; RET; NTRK1; CSF1R; PDGFRA	7
Brigatinib	EGFR; ABL1; IGF1R; INSR; MET; ERBB2	6
Sorafenib	PDGFRB; KIT; KDR; FGFR1; RET; FLT1	6
Sunitinib	PDGFRB; FLT1; KDR; KIT; CSF1R; PDGFRA	6
Nintedanib	FLT1; KDR; FGFR1; LCK; LYN; SRC	6
Pazopanib	FLT1; KDR; PDGFRA; PDGFRB; KIT	5
Midostaurin	PRKCA; KDR; KIT; PDGFRA; PDGFRB	5
Resveratrol	ITGA5; ITGB3; SNCA; ESR1; AKT1	5
Diethylstilbestrol	ESR1; ESRRG; ESR2; ESRRA	4
Tofacitinib	TYK2; JAK2; JAK1; JAK3	4
Lenvatinib	FLT1; KDR; FGFR1; KIT	4
Foreskin fibroblast (neonatal)	FLT1; CSF2RA; PDGFRB; TGFB1	4
Baricitinib	JAK1; JAK2; PTK2B; JAK3	4
Foreskin keratinocyte (neonatal)	EGFR; CSF2RA; PDGFRA; TGFB1	4
Bosutinib	ABL1; LYN; SRC	3
Estradiol valerate	ESR1; ESR2; ESRRG	3

## Discussion

### Putative targets from our computational framework

Among the 187 putative targets from our analysis (see Supplementary Table 1), we investigated the top ranked genes and found that several of these genes play an important role in the mechanism of Alzheimer’s disease.

For example, the first ranked putative target, DLG4, encodes PSD95, which is a key protein for synaptic plasticity that is downregulated in under aged patients as well as patients with Alzheimer’s disease. Recently, Bustos et al. demonstrated that epigenetic editing of DLG4/PSD95 ameliorates cognitions in model mice with Alzheimer’s disease [48]. Thus, epigenetic editing of DLG4 may serve as a novel therapy for rescuing cognitive impairment induced by Alzheimer’s disease.

EGFR is the third ranked putative target and is frequently upregulated in certain cancers. By employing an amyloid- *β*-expressing fruit fly model, Wang et al. demonstrated that the upregulation of EGFR causes memory impairment [49]. Furthermore, they administered several EGFR inhibitors (e.g., erlotinib and gefitinib) to transgenic fly and a mouse model of Alzheimer’s disease and found that the inhibitors prevented memory loss in both animal models. Based on these findings, they suggested that EGFR may be a therapeutic target for the treatment of amyloid- *β*-induced memory impairment.

RAC1, the sixth ranked putative target, is a small signaling GTPase, that controls different cellular processes, including cell growth, cellular plasticity, and inflammatory responses. Inhibition of RAC1 downregulates amyloid precursor protein (APP) and amyloid- *β* through regulation of the APP gene in hippocampal primary neurons [50]. RAC1 inhibitors can prevent cell death caused by amyloid- *β*42 in primary neurons of the hippocampus and those of the entorhinal cortex [51]. Furthermore, based on an analysis of the protein-domain interaction network and experiments using drosophila genetic models, Kikuchi et al. demonstrated that RAC1 is a hub gene in the network and thus causes age-related alterations in behavior and neuronal degenerations [52]. The RAC1 gene could be a potential therapeutic target for preventing amyloid- *β*-induced neuronal cell death in Alzheimer’s disease.

Spleen tyrosine kinase (SYK), the fourth ranked potential target, could modulate the accumulation of amyloid- *β* and hyperphosphorylation of Tau protein, which is associated with Alzheimer’s disease [53]. Nilvadipine, an antagonist of the L-type calcium channel (LCC), inhibits the accumulation of amyloid- *β*; however, this does not occur because of LCC inhibition, but rather other mechanisms. Paris et al. demonstrated that the down-regulation of SYK exerts an effect that is similar to an enantiomer of Nilvadipine ((-)-nilvadipine) for the clearance of amyloid- *β* and reduction of Tau hyperphosphorylation [53]. Schweig et al. demonstrated that in mice with overexpressing amyloid- *β*, SYK activation occurred in the microglia. Further, neurite degeneration was found to increase because of the association between amyloid- *β* plaques and aging [54]. These researchers also demonstrated that in mice overexpressing Tau, SKY was activated in the microglia while misfolded and hyperphosphorylated Tau was accumulated in the hippocampus and cortex. Schweig et al. demonstrated that SYK inhibition induces Tau reduction in an autophagic manner [55]. Moreover, they demonstrated that SYK acts as an upstream target in the mTOR pathway and its inhibition induces Tau degradation by decreasing the activation of mTOR pathway.

The 5th ranked putative target, PTK2B, is a key gene in the mediation of synaptic dysfunction induced by amyloid- *β* in Alzheimer’s disease [56]. Salazar et al. demonstrated that in a transgenic mice model of Alzheimer’s disease, PTK2B deletion improves deficits in memory and learning functions as well as synaptic loss [56].

Although SOCS1 is the 78th ranked putative target, it modulates cytokine responses by suppressing JAK/STAT signaling to control inflammation in the CNS (central nerve system) [57]. Thus, SOCS1 may be a key therapeutic modulator in Alzheimer’s disease.

GWAS and other sequencing technologies have identified over 20 genes that modify Alzheimer’s disease risk. We obtained 29 genes listed in [58] and compared them with our 187 genes. PTK2B and INPP5D were listed as the overlap between the two gene sets. While as mentioned above, PTK2B is the 5th ranked strong candidate gene, INPP5D was the 68th ranked putative gene in the set of our 187 genes. INPP5D (Inositol Polyphosphate-5-Phosphatase D) is selectively expressed in brain microglia and likely a crucial player in Alzheimer’s disease pathophysiology. Tsai et al. reported that INPP5D expression was upregulated in late-onset Alzheimer’s disease and positively correlated with amyloid plaque density [59].

Collectively, these findings indicate that our computational framework could successfully identify key genes that may be novel target candidates for Alzheimer’s disease.

### Promising repositionable drugs for Alzheimer’s disease

In our computational drug repositioning analysis, our method predicted that tamoxifen (the second ranked candidate, see Table 2), an FDA-approved estrogen receptor modulator for the treatment of hormone-receptor-positive breast cancer patients, could serve as a potential drug target for Alzheimer’s disease. As mentioned in Wise PM [60], estrogen therapy could protect neuronal cells from cell death by modulating the expression of key genes that inhibit the apoptotic cell death pathway. Based on a nation-wide cohort study in Taiwan, Sun et al. reported that patients with long-term use of tamoxifen exhibited a reduced risk of dementia [61].

Our method also predicted that bosutinib (the nineteenth ranked target), an FDA-approved tyrosine-kinase-inhibitor (TKI) drug (Bcr-Abl kinase inhibitor) for the treatment of Philadelphia chromosome-positive (Ph+) chronic myelogenous leukemia, may be a repositionable drug for Alzheimer’s disease (see Table 2). Lonskaya et al. reported that Bosutinib combined with nilotinib systematically modulates with immune system in the CNS by inhibiting the non-receptor tyrosine kinase, Abl, to remove amyloid and decrease neuroinflammation [62]. Such findings indicates that TKIs, especially bosutinib, could be potential repositionable drugs for the treatment of early stage Alzheimer’s disease.

Among the predicted repositionable candidates, 19 are immunosuppressive agents. These 19 candidates may include promising repositionable drugs for Alzheimer’s disease; this is because of the important role played by inflammation in the mechanisms of Alzheimer’s disease. Among the 19 candidates, dasatinib (the fourth ranked compound) may be the most promising candidate. Recently, Zhang et al. reported that senolytic therapy (a combination of dasanitib and quercetin) could reduce the production of proinflammatory cytokine and alleviate deficits of cognitive functions in Alzheimer’s disease mouse models, via the selective removal of senescent oligodendrocyte progenitor cells [63, 64]. Furthermore, the combined therapy of dasatinib and quercetin is now registered in a clinical trial (ClinicalTrials.gov Identifier: NCT04063124).

One limitation of our method was that the process of identifying the putative target genes was dependent on the drug taget gene database (i.e., the DrugBank in this research). This means that there is a possibility of bias in the known target genes because the DrugBank contains the existing therapeutic drugs and compounds which may have failed the clinical trials. However, we could overcome this limitation by adding new drug and target relationships, such as tau targeting compounds.

## Conclusions

In this study, we developed a deep autoencoder-based computational framework and applied it to prioritize putative target genes for Alzheimer’s disease. The method identified key genes (e.g., DLG4, EGFR, RAC1, SYK, PTK2B, SOCS1) associated with the disease mechanisms. Furthermore, by using the putative targets, we successfully inferred promising repositionable candidate-compounds (e.g., tamoxifen, bosutinib, dasatinib) for Alzheimer’s disease. Our method could be a powerful tool for inferring potential repositionable drugs, especially those that could be used to treat Alzheimer’s disease. Notably, our computational framework can be easily applied to the investigation of novel potential therapeutic targets and repositioning compounds for any disease. Accordingly, we anticipate that our method will be used by large pharmaceutical companies that house large volumes of their own non-public data.

## Supplementary Information

**Additional file 1** The original data of Fig. 2. Rows and columns represent the names of features in the low-dimensional latent space and names of the network metrics, respectively. The numeric value in a cell represents Spearman’s correlation coefficient between a given low-dimensional feature and a given network metric (i.e., the correlation coefficient between the feature “Dimension 1” and the network metric “outdegree” is 0.67). Darker red (blue) indicates a higher (lower) correlation coefficient. Dimensions that are zero for all genes are denoted as n.a.

**Additional file 2** A list of potential therapeutic targets for Alzheimer’s disease.

**Additional file 3** A list of all candidate repositionable compounds for Alzheimer’s disease.

## Data Availability

Documentation and source code are available on the author’s Github site[65]. Declarations

## Abbreviations

PIN: Protein-protein interaction network
AI: Artificial intelligence
PCA: Principle component analysis
ReLU: Rectified linear unit
NND: Nearest neighbor degree
ORA: Over-representation analysis
