# Supplementary material for: Artificial intelligence-based computational framework for drug-target prioritization and inference of novel repositionable drugs for Alzheimer’s disease
Source: Alzheimers Res Ther. 2021 May 3;13:92. doi: 10.1186/s13195-021-00826-3 (PMC8091739; doi:10.1186/s13195-021-00826-3)
Supplement: Supplementary file 1 — Additional file 1 The original data of Fig. 2. Rows and columns represent the names of features in the low-dimensional latent space and names of the network metrics, respectively. The numeric value in a cell represents Spearman’s correlation coefficient between a given low-dimensional feature and a given network metric (i.e., the correlation coefficient between the feature “Dimension 1” and the network metric “outdegree” is 0.67). Darker red (blue) indicates a higher (lower) correlation coefficient. Dimensions that are zero for all genes are denoted as n.a. [file 13195_2021_826_MOESM1_ESM.pdf]

Latent space	Centrality measures					Bow-tie analysis			Controllability	Other metrics	
	In degree	Out degree	Betweenness	Closeness	Page rank	Input layer	Output layer	Core layer	Indispensable	Cluster coefficient	NND
dimension 0	0.181	0.351	0.177	0.155	0.240	0.063	0.041	-0.085	0.279	0.158	-0.086
dimension 1	0.271	0.670	0.420	0.499	0.372	0.176	-0.284	0.043	0.360	0.051	-0.018
dimension 2	0.034	0.438	0.202	0.544	0.056	0.356	-0.183	-0.196	0.254	-0.151	-0.194
dimension 3	0.225	0.530	0.303	0.352	0.261	0.168	-0.121	-0.065	0.356	0.106	-0.145
dimension 4	-0.172	-0.006	-0.138	0.242	-0.192	0.316	0.114	-0.370	0.022	-0.179	-0.237
dimension 5	0.332	0.482	0.315	0.112	0.454	-0.058	-0.075	0.110	0.350	0.274	0.021
dimension 6	n.a.	n.a.	n.a.	n.a.	n.a.	n.a.	n.a.	n.a.	n.a.	n.a.	n.a.
dimension 7	n.a.	n.a.	n.a.	n.a.	n.a.	n.a.	n.a.	n.a.	n.a.	n.a.	n.a.
dimension 8	0.289	0.609	0.364	0.376	0.381	0.141	-0.174	-0.002	0.390	0.143	-0.068
dimension 9	0.117	0.596	0.335	0.653	0.233	0.319	-0.369	-0.030	0.253	-0.170	-0.009
dimension 10	0.295	0.414	0.264	0.094	0.426	-0.044	-0.056	0.082	0.319	0.252	0.023
dimension 11	0.089	0.515	0.283	0.558	0.085	0.321	-0.226	-0.131	0.274	-0.120	-0.183
dimension 12	n.a.	n.a.	n.a.	n.a.	n.a.	n.a.	n.a.	n.a.	n.a.	n.a.	n.a.
dimension 13	0.250	0.457	0.286	0.196	0.243	0.082	-0.067	-0.024	0.347	0.199	-0.166
dimension 14	0.110	0.560	0.313	0.602	0.154	0.319	-0.269	-0.100	0.275	-0.129	-0.117
dimension 15	n.a.	n.a.	n.a.	n.a.	n.a.	n.a.	n.a.	n.a.	n.a.	n.a.	n.a.
dimension 16	-0.034	0.229	0.112	0.326	-0.191	0.307	-0.105	-0.204	0.156	-0.107	-0.344
dimension 17	-0.309	-0.064	-0.191	0.372	-0.361	0.410	0.084	-0.437	-0.079	-0.367	-0.258
dimension 18	n.a.	n.a.	n.a.	n.a.	n.a.	n.a.	n.a.	n.a.	n.a.	n.a.	n.a.
dimension 19	0.321	0.603	0.396	0.288	0.404	0.035	-0.187	0.104	0.367	0.183	-0.027
dimension 20	n.a.	n.a.	n.a.	n.a.	n.a.	n.a.	n.a.	n.a.	n.a.	n.a.	n.a.
dimension 21	0.377	0.682	0.485	0.377	0.566	0.001	-0.354	0.252	0.339	0.143	0.182
dimension 22	0.409	0.647	0.506	0.242	0.471	-0.068	-0.319	0.292	0.366	0.239	0.057
dimension 23	n.a.	n.a.	n.a.	n.a.	n.a.	n.a.	n.a.	n.a.	n.a.	n.a.	n.a.
dimension 24	0.080	0.360	0.171	0.370	0.109	0.257	-0.153	-0.126	0.234	-0.038	-0.153
dimension 25	0.296	0.522	0.312	0.228	0.412	0.037	-0.084	0.028	0.359	0.209	-0.013
dimension 26	0.338	0.617	0.403	0.339	0.593	-0.003	-0.293	0.210	0.311	0.129	0.228
dimension 27	0.216	0.297	0.211	0.144	0.303	-0.012	-0.074	0.064	0.225	0.155	0.107
dimension 28	-0.213	0.061	-0.078	0.385	-0.313	0.408	0.015	-0.385	0.024	-0.286	-0.326
dimension 29	0.264	0.571	0.341	0.391	0.502	0.073	-0.242	0.104	0.285	0.064	0.181
dimension 30	0.277	0.559	0.320	0.343	0.513	0.070	-0.208	0.083	0.312	0.110	0.144
dimension 31	0.294	0.642	0.380	0.425	0.476	0.133	-0.273	0.073	0.364	0.097	0.055
dimension 32	0.412	0.772	0.551	0.422	0.534	0.045	-0.416	0.257	0.395	0.163	0.090
dimension 33	0.255	0.573	0.341	0.361	0.298	0.148	-0.138	-0.035	0.369	0.124	-0.121
dimension 34	0.023	0.449	0.220	0.584	0.048	0.350	-0.213	-0.169	0.216	-0.194	-0.157
dimension 35	-0.162	-0.022	-0.112	0.145	-0.325	0.272	0.165	-0.365	0.034	-0.118	-0.364
dimension 36	0.157	-0.024	0.007	-0.324	0.200	-0.242	0.195	0.084	0.118	0.299	0.034
dimension 37	0.365	0.466	0.323	0.068	0.551	-0.124	-0.106	0.190	0.330	0.296	0.127
dimension 38	0.147	0.500	0.266	0.496	0.373	0.192	-0.270	0.014	0.219	-0.084	0.141
dimension 39	0.331	0.574	0.355	0.274	0.557	0.007	-0.213	0.145	0.339	0.178	0.144
dimension 40	0.079	0.358	0.177	0.331	0.001	0.244	-0.026	-0.203	0.260	0.001	-0.284
dimension 41	0.201	0.309	0.209	0.045	0.134	0.009	0.002	-0.006	0.276	0.216	-0.193
dimension 42	0.119	0.541	0.290	0.558	0.182	0.299	-0.235	-0.106	0.294	-0.097	-0.117
dimension 43	0.375	0.632	0.412	0.286	0.623	-0.030	-0.263	0.215	0.346	0.194	0.202
dimension 44	0.013	0.372	0.198	0.461	-0.074	0.306	-0.149	-0.172	0.193	-0.141	-0.254
dimension 45	0.184	0.524	0.353	0.409	0.093	0.221	-0.252	-0.020	0.321	0.030	-0.234
dimension 46	-0.348	-0.230	-0.301	0.225	-0.410	0.370	0.144	-0.443	-0.155	-0.337	-0.235
dimension 47	0.074	0.421	0.175	0.475	0.187	0.287	-0.125	-0.174	0.258	-0.083	-0.090
dimension 48	-0.294	-0.227	-0.237	0.059	-0.542	0.279	0.181	-0.382	-0.120	-0.204	-0.389
dimension 49	0.246	0.602	0.341	0.444	0.408	0.171	-0.256	0.025	0.347	0.053	0.015
dimension 50	-0.035	0.345	0.134	0.512	-0.052	0.363	-0.127	-0.242	0.189	-0.199	-0.235
dimension 51	n.a.	n.a.	n.a.	n.a.	n.a.	n.a.	n.a.	n.a.	n.a.	n.a.	n.a.
dimension 52	n.a.	n.a.	n.a.	n.a.	n.a.	n.a.	n.a.	n.a.	n.a.	n.a.	n.a.
dimension 53	n.a.	n.a.	n.a.	n.a.	n.a.	n.a.	n.a.	n.a.	n.a.	n.a.	n.a.
dimension 54	0.107	0.486	0.273	0.492	0.098	0.307	-0.248	-0.103	0.289	-0.072	-0.198
dimension 55	0.286	0.564	0.335	0.304	0.410	0.074	-0.124	0.023	0.360	0.163	-0.005
dimension 56	0.261	0.167	0.133	-0.204	0.413	-0.257	0.099	0.166	0.192	0.318	0.161
dimension 57	0.238	0.526	0.281	0.357	0.417	0.127	-0.156	-0.005	0.325	0.094	0.040
dimension 58	0.022	0.022	0.022	0.014	0.022	-0.008	-0.005	0.010	0.024	0.021	-0.005
dimension 59	-0.221	-0.321	-0.260	-0.131	-0.393	0.118	0.224	-0.266	-0.142	-0.076	-0.230
dimension 60	n.a.	n.a.	n.a.	n.a.	n.a.	n.a.	n.a.	n.a.	n.a.	n.a.	n.a.
dimension 61	0.250	0.260	0.180	0.020	0.508	-0.136	-0.081	0.180	0.170	0.185	0.274
dimension 62	-0.257	-0.071	-0.136	0.229	-0.478	0.366	0.069	-0.383	-0.051	-0.247	-0.406
dimension 63	0.358	0.469	0.336	0.103	0.530	-0.086	-0.163	0.197	0.322	0.272	0.116
dimension 64	0.004	0.339	0.126	0.443	0.003	0.315	-0.047	-0.255	0.215	-0.122	-0.222
dimension 65	0.190	0.619	0.344	0.565	0.324	0.264	-0.292	-0.035	0.338	-0.043	-0.029
dimension 66	0.113	0.277	0.106	0.179	0.160	0.130	0.062	-0.161	0.244	0.096	-0.134
dimension 67	-0.307	-0.243	-0.282	0.067	-0.476	0.293	0.232	-0.433	-0.106	-0.214	-0.360
dimension 68	-0.022	0.390	0.176	0.564	-0.043	0.371	-0.173	-0.216	0.186	-0.218	-0.212
dimension 69	-0.007	0.371	0.162	0.515	-0.019	0.373	-0.200	-0.199	0.201	-0.179	-0.218
dimension 70	0.262	0.546	0.385	0.295	0.221	0.092	-0.227	0.081	0.336	0.134	-0.152
dimension 71	-0.218	0.085	-0.063	0.432	-0.296	0.423	-0.039	-0.360	0.019	-0.319	-0.302
dimension 72	-0.091	0.225	0.018	0.478	0.055	0.333	-0.119	-0.224	0.079	-0.255	-0.028
dimension 73	0.107	0.385	0.151	0.373	0.287	0.193	-0.081	-0.121	0.234	-0.021	0.023
dimension 74	-0.107	-0.305	-0.261	-0.255	-0.010	-0.086	0.251	-0.101	-0.112	0.030	0.078
dimension 75	0.009	0.379	0.147	0.511	0.048	0.353	-0.128	-0.232	0.231	-0.152	-0.188
dimension 76	0.130	0.530	0.286	0.563	0.333	0.241	-0.323	0.007	0.222	-0.131	0.103
dimension 77	0.116	-0.042	0.004	-0.296	0.035	-0.178	0.175	0.041	0.115	0.267	-0.114
dimension 78	n.a.	n.a.	n.a.	n.a.	n.a.	n.a.	n.a.	n.a.	n.a.	n.a.	n.a.
dimension 79	n.a.	n.a.	n.a.	n.a.	n.a.	n.a.	n.a.	n.a.	n.a.	n.a.	n.a.
dimension 80	0.123	0.514	0.248	0.531	0.279	0.275	-0.217	-0.099	0.285	-0.078	-0.020
dimension 81	0.403	0.633	0.428	0.242	0.649	-0.078	-0.253	0.252	0.350	0.230	0.219
dimension 82	0.353	0.476	0.416	0.077	0.318	-0.138	-0.208	0.277	0.266	0.261	0.036
dimension 83	-0.040	0.336	0.120	0.522	-0.016	0.382	-0.172	-0.228	0.181	-0.209	-0.195
dimension 84	0.185	0.621	0.397	0.561	0.216	0.243	-0.353	0.031	0.287	-0.068	-0.074
dimension 85	-0.210	0.081	-0.045	0.390	-0.354	0.405	-0.017	-0.358	0.023	-0.288	-0.356
dimension 86	-0.007	0.023	-0.001	0.017	0.001	0.009	-0.006	-0.003	-0.009	-0.008	-0.007
dimension 87	0.028	0.482	0.244	0.615	0.045	0.374	-0.271	-0.149	0.223	-0.208	-0.163
dimension 88	0.067	0.094	0.073	0.023	0.085	-0.039	-0.025	0.053	0.019	0.043	0.048
dimension 89	0.085	0.121	0.091	0.092	0.091	0.030	-0.027	-0.008	0.092	0.051	-0.017
dimension 90	-0.021	0.271	0.062	0.432	0.141	0.282	-0.130	-0.169	0.130	-0.174	-0.003
dimension 91	n.a.	n.a.	n.a.	n.a.	n.a.	n.a.	n.a.	n.a.	n.a.	n.a.	n.a.
dimension 92	0.397	0.698	0.466	0.352	0.642	-0.008	-0.332	0.243	0.362	0.178	0.210
dimension 93	n.a.	n.a.	n.a.	n.a.	n.a.	n.a.	n.a.	n.a.	n.a.	n.a.	n.a.
dimension 94	0.162	0.558	0.294	0.530	0.345	0.235	-0.267	-0.027	0.288	-0.062	0.041
dimension 95	0.465	0.776	0.566	0.363	0.660	-0.035	-0.420	0.333	0.393	0.217	0.196
dimension 96	-0.190	-0.022	-0.132	0.218	-0.296	0.323	0.117	-0.378	0.026	-0.181	-0.336
dimension 97	n.a.	n.a.	n.a.	n.a.	n.a.	n.a.	n.a.	n.a.	n.a.	n.a.	n.a.
dimension 98	0.027	0.484	0.242	0.618	0.068	0.376	-0.305	-0.128	0.219	-0.219	-0.136
dimension 99	0.060	0.324	0.115	0.379	0.285	0.188	-0.139	-0.076	0.157	-0.094	0.108
